# Comparison of Efficacy of Beractant (SURVANTA®) and Poractant Alfa (CUROSURF®) in Preterm Infants With Respiratory Distress Syndrome in Tawam Hospital, Al Ain, UAE: A Retrospective Study

**DOI:** 10.7759/cureus.74790

**Published:** 2024-11-29

**Authors:** Fahad Butt, Huda Mohamad, Nour Abusallout, Alaa Zaineh, Feryal El khatib, Sarah Abdulrahman, Sara Hamwi, Mohamed Rahmani, Mustafa Alabdullatif

**Affiliations:** 1 Neonatology, Tawam Hospital, Al Ain, ARE; 2 Pediatrics, Tawam Hospital, Al Ain, ARE; 3 Pediatrics, Tawam hospital, Al Ain, ARE; 4 Biological Sciences, Khalifa University, Abu Dhabi, ARE

**Keywords:** beractant, bronchopulmonary dysplasia, poractant, preterm infants, respiratory distress syndrome, surfactant therapy

## Abstract

Introduction

Respiratory distress syndrome (RDS) is a leading cause of morbidity and mortality among preterm infants, necessitating effective treatment strategies. This study compared the efficacy of Beractant (SURVANTA®) to Poractant alfa (CUROSURF®) in treating RDS in preterm infants admitted to Tawam Hospital in the UAE.

Methodology

This retrospective study included preterm infants from 23+0 to 36+6 weeks of gestation with a diagnosis of RDS and treatment by Beractant or Poractant alfa within 48 hours of life between January 2020 and March 2023. Data collected from electronic medical records of Tawam Hospital include infant and maternal demographics, primary outcome parameters, such as bronchopulmonary dysplasia (BPD) and/or mortality, and secondary outcome parameters, such as surfactant redosing, air leak syndrome, and other complications.

Results

A total of 258 infants met the inclusion criteria: 178 were treated with Beractant, and 80 were treated with Poractant alfa. After adjusting the confounding factors, the occurrence of bronchopulmonary dysplasia (BPD) was not statistically significant, showing rates of 68.7% (n=46) in the Poractant group and 47.5% (n=75) in the Beractant group (p=0.71). Likewise, there was no significant difference in mortality rates between the two groups, with 22.5% (n=18) in the Poractant group and 11.8% (n=21) in the Beractant group (p=0.33). Furthermore, the combined incidence of BPD or mortality was also not statistically significant, recorded at 53.4% (n=95) for the Beractant group compared to 73.8% (n=59) for the Poractant group (p=0.93).

However, the need for surfactant redosing and air leak syndrome was significantly lower in the Poractant group compared to the Beractant group, 26.2% (n=21) vs 45.5% (n=81), p < 0.00001 and 8.8% (n=7) vs 14.6% (n=26), p = 0.05, respectively. There was no difference in the incidence of other outcomes such as pulmonary hemorrhage, periventricular leukomalacia (PVL), intraventricular hemorrhage (IVH), necrotizing enterocolitis (NEC), significant patent ductus arteriosus (PDA), and retinopathy of prematurity (ROP) that required treatment.

Conclusion

There was no significant difference in the rates of bronchopulmonary dysplasia or mortality between Poractant alfa (CUROSURF®) and Beractant (SURVANTA®) in preterm infants suffering from respiratory distress syndrome. Poractant alfa (CUROSURF®) showed a reduced need for surfactant redosing and a lower incidence of air leak syndrome. However, the rates of other outcomes, including significant patent ductus arteriosus (PDA), intraventricular hemorrhage (IVH), retinopathy of prematurity (ROP), periventricular leukomalacia (PVL), and necrotizing enterocolitis (NEC), were comparable in both treatment groups. Further randomized prospective studies are necessary to evaluate these types of surfactants and investigate their efficacy as well as both short- and long-term outcomes.

## Introduction

Respiratory distress syndrome (RDS) is considered one of the common diseases affecting premature infants, with an incidence of approximately 80% in those born at or before 28 weeks of gestational [1]. The mechanism of this disease is mainly due to alveolar surfactant deficiency, which leads to reduced alveolar surface tension, microatelectasis, and decreased lung volume [2]. Surfactant replacement therapy has revolutionized managing RDS and forms one of the cornerstones in neonatal care. Using exogenous surfactants significantly improved survival and reduced chronic lung diseases in preterm infants to a greater degree [3]. Natural surfactants are preferred over synthetic ones due to their association with lower rates of adverse outcomes [4,5].

There are differences in composition and effectiveness among animal-sourced surfactants. Beractant (SURVANTA®) and Poractant alfa (CUROSURF®) are among the most popular natural surfactants used in neonatal care [6]. Beractant (SURVANTA®) is derived from bovine lung extract. It contains a higher proportion of total neutral lipids and a lower proportion of polyunsaturated fatty acid-containing phospholipids (PUFA-PLs) compared to Poractant alfa (CUROSURF®). It contains 25 mg/mL phospholipids, with an initial 4 mL/kg dose. Poractant alfa (CUROSURF®), on the other hand, is derived from porcine minced lung extract. It contains a larger amount of phospholipids with a smaller size distribution and a larger amount of plasmalogens compared to Beractant (SURVANTA®). It contains 75 mg/mL phospholipids, with an initial 2.5 mL/kg dose. These variations in the components and the recommended doses could explain some differences in the clinical effectiveness [7,8]. Many studies have compared both types of surfactants against each other; however, results have varied in outcomes like mortality, morbidities, and redosing [9-11]. Evaluating these differences in the efficacy of both types is vital for optimizing treatment protocols in the NICU.

Our hospital used Beractant (SURVANTA®) until January 2022. Since then, we have been using Poractant alfa (CUROSURF®). Our retrospective study compared the effectiveness of Beractant and Poractant alfa in RDS management among preterm infants admitted to Tawam Hospital over the period 2020-2023. In this study, we aim to determine the most effective surfactant therapy to improve patient outcomes and resource utilization within our clinical settings.

## Materials and methods

Study design

We compared outcomes retrospectively among neonates who received either Beractant or Poractant alfa. Data was collected by reviewing the electronic medical records of Tawam Hospital and entering them into a data collection sheet. This study included various demographic factors related to both infants and mothers, such as gestational age, birth weight, gender, Apgar scores, antenatal corticosteroid therapy, and other maternal and neonatal morbidities. We categorized the study population into two groups based on the type of surfactant administered: Beractant or Poractant alfa. Outcomes were evaluated on subgroups of individuals under 32 weeks of gestation.

Definitions

Bronchopulmonary dysplasia (BPD) was diagnosed and classified according to Jensen criteria [12]. Intraventricular hemorrhage (IVH) was diagnosed based on cranial ultrasound scanning during the NICU stay, classified using Papile'sscale [13]. Periventricular leukomalacia (PVL) was defined as periventricular echogenicity on a cranial ultrasound scan. The Standard International Classification defined retinopathy of prematurity (ROP) [14]. Necrotizing enterocolitis (NEC) was diagnosed according to the modified Bell's criteria at stage two or higher [15]. Sepsis was defined as positive blood culture growth (peripheral or central line) during NICU stay. Early onset sepsis was defined as the positive blood culture collected in the first 72 hours of life. Patent ductus arteriosus (PDA) was considered significant when medical or surgical treatment was required. Pulmonary air leak syndrome was defined as the occurrence of any of the following: pneumothorax, pneumomediastinum, pneumopericardium, subcutaneous emphysema, or pulmonary interstitial emphysema. Pulmonary hemorrhage was defined as bright red blood spouted out of the endotracheal tube with typical chest radiographic findings and rapid deterioration of the patient's condition. The Clinical Risk Index for Babies (CRIB) score is a risk-adjustment tool used in neonatal intensive care. It provides a simplified scoring system that avoids the potential problems of early treatment bias [16].

Settings and inclusion criteria

Tawam Hospital is one of the largest tertiary teaching hospitals within Abu Dhabi Health Services Company in the United Arab Emirates (UAE). It has a Level 3+ NICU with a capacity of 51 beds and manages about 3,000 deliveries yearly. Between January 2020 and March 2023, we enrolled preterm infants born at our facility, with gestational ages from 23 weeks and 0 days to 36 weeks and six days, who were treated with either Beractant (SURVANTA®) or poractant alfa (CUROSURF®) via endotracheal tube.

Exclusion criteria

We excluded the infants who had any of the following: Any major congenital abnormalities, those who were not delivered at Tawam Hospital, or any infant who passed away within 24 hours of birth.

Primary outcomes

The primary outcomes of the study were to evaluate the incidence and mortality rates of bronchopulmonary dysplasia (BPD) during NICU stays in the two studied groups.

Secondary outcomes

Secondary outcomes were to assess the need for surfactant redosing, the occurrence of pulmonary air leaks (such as pneumothorax, pneumomediastinum, or pulmonary interstitial emphysema), and pulmonary hemorrhage. Additionally, we evaluated the incidence of several conditions, including significant PDA, IVH, PVL, ROP, NEC, and sepsis.

Statistical methods

Descriptive statistics (frequency, percent, mean, and standard deviation (SD)) were reported for the demographic variables. Maternal and infant parameters were compared using Pearson's Chi-squared test and student t-test for categorical and numerical variables, respectively. Primary and secondary outcomes were analyzed between the two groups (Poractant alfa and Beractant) using a multivariate logistic regression model adjusted for confounders. Adjusted odds ratios (ORs) and 95% confidence intervals (CIs) were calculated for all outcomes. Statistics were analyzed using BlueSky statistics 10.3.2.

## Results

**Figure 1:**
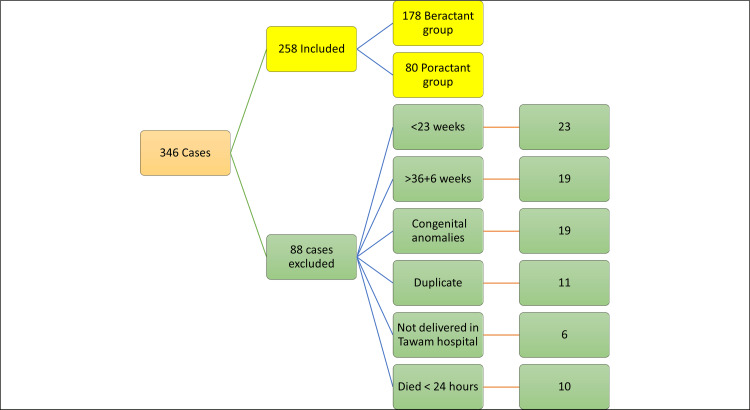
Flowchart of the study

A review of 346 cases was conducted, with 258 cases qualifying for inclusion in the study. The other 88 cases were excluded for several reasons, such as gestational age being below 23 weeks or above 36+6 weeks, having significant congenital anomalies, duplicated records, being born outside Tawam Hospital, or passing away within 24 hours of birth. The study population was categorized into two groups: Group 1 had 178 infants who were given Beractant, while Group 2 had 80 infants who received Poractant alfa (Figure 1).

Demographic characteristics of the whole population

In comparison to both groups, a greater percentage of neonates in the poractant group had gestational ages between 23+0 weeks and 27+6 weeks, with 57.5% (n=46) in this group versus 34.8% (n=62) in the beractant group (p=0.006). Furthermore, the poractant group showed a significantly lower mean birth weight compared to the beractant group, with weights of 1306 grams vs. 1406 grams, respectively (p<0.001). The incidence of chorioamnionitis was also higher in the poractant group, at 15% (n=12) compared to 4.5% (n=8) in the beractant group (p=0.004). We also noted a higher mean CRIB II score in the poractant group, 10.1 vs 7.5 in the beractant group. No other major differences in demographic characteristics were noted, as outlined in Table 1.

**Table 1:** Infant and maternal demographic characteristics of the whole cohort P-value < 0.05 is considered significant. Student t-test was used for numerical variables, and chi-square test was used for categorical variables to calculate the p-value. ^t test value, *Pearson Chi-square test value GA: Gestational Age; SD: standard deviation; BW: birth weight; g: grams; CRIB: Clinical Risk Index for Babies

Variable	Group 1: Beractant (N=178)	Group 2: Poractant alfa (N=80)	Statistical Test Value	p-value
Gestational age at birth				
23+0 – 27+6 weeks, n/N (%)	62/178 (34.8)	46/80 (57.5)	12.34*	0.006
28+0 – 31+6 weeks, n/N (%)	64/178 (36.0)	20/80 (25.0)		
32+0 – 33+6 weeks, n/N (%)	23/178 (12.9)	8/80 (10.0)		
34+0 – 36+6 weeks, n/N (%)	29/178 (16.3)	6/80 (7.5)		
Overall GA (weeks), mean (SD)	29 (3.4)	27 (3.6)	4.52^	<0.001
Birth weight				
≤ 750 (g), n/N (%)	28/178 (15.7)	31/80 (38.8)	19.23*	< 0.001
751 - 999 (g), n/N (%)	30/178 (16.9)	14/80 (17.5)		
1000 -1499 (g), n/N (%)	55/178 (30.9)	18/80 (22.5)		
1500 – 2499 (g), n/N (%)	54/178 (30.3)	16/80 (20.0)		
≥2500 (g), n/N (%)	11/178 (6.2)	1/80 (1.2)		
Overall BW (g), mean (SD)	1406 (671)	1306 (666)	3.81^	<0.001
Male, n/N (%)	103/178 (57.9)	55/80 (68.8)	2.75*	0.097
CRIB II score, mean (SD)	7.5 (4)	10.1 (4.7)	4.8^	<0.001
Apgar score				
1 min, mean (SD)	5.4 (2)	5.2 (2)	0.84^	0.427
5 min, mean (SD)	7.4(1)	7.1 (2)	1.84^	0.055
10 min, mean (SD)	8.5 (1)	8.2 (1)	2.17^	0.031
Mode of delivery				
Caesarean section, n/N (%)	139/178 (78.1)	55/80 (68.8)	2.58*	0.108
Vaginal delivery, n/N (%)	39/178 (21.9)	25/80 (31.2)		
Antenatal Steroids				
No or Inadequate course, n/N (%)	92/178 (51.7)	38/80 (47.5)	0.38*	0.534
Complete course, n/N (%)	86/178 (48.3)	42/80 (52.5)		
Antenatal Antibiotics				
No or Inadequate, n/N (%)	128/178 (71.9)	60/80 (75)	0.26*	0.606
Adequate, n/N (%)	50/178 (28.1)	20/80 (25)		
Antenatal Magnesium Sulphate, n/N (%)	64/178 (36)	32/80 (40)	0.38*	0.534
Postnatal steroid therapy, n/N (%)	17/178 (9.6)	6/80 (7.5)	0.23*	0.593
Positive Maternal Group B streptococcus, n/N (%)	25/102 (24.5)	13/36 (36.1)	1.79*	0.180
Chorioamnionitis, n/N (%)	8/177 (4.5)	12/80 (15)	8.43*	0.004
Premature rupture of membranes, n/N (%)	43/177 (24.3)	22/79 (27.8)	0.36*	0.546
Maternal hypertension n/N (%)	29/173 (16.8)	17/74 (23)	1.31*	0.251
Maternal diabetes mellitus, n/N (%)	63/171 (36.8)	27/75 (36)	0.01*	0.9
Early onset sepsis, n/N (%)	5/178 (2.8)	3/80 (3.8)	0.15*	0.687

Demographic characteristics of the neonates <32 weeks of gestation

In this subgroup of neonates below 32 weeks of gestation, those treated with poractant had notably lower mean gestational ages and birth weights compared to those who received Beractant. Specifically, the mean gestational age was 26 weeks for the Poractant group and 28 weeks for the beractant group (p<0.001). Additionally, the mean birth weight was 875 grams in the Poractant group compared to 1070 grams in the Beractant group (p<0.001). Furthermore, the incidence of chorioamnionitis and the CRIB II score were higher in the poractant group than in the beractant group (Table 2).

**Table 2:** Infant and maternal demographic characteristics of the neonates below 32 weeks of gestation ^t test value, *Pearson Chi-square test value p-value < 0.05 is considered significant. Linear model ANOVA was used for numerical variables, and the Chi-square test was used for categorical variables to calculate the p-value GA: Gestational Age; SD: standard deviation; BW: birth weight; g: grams

Variable	Group 1: Beractant (N=126)	Group 2: Poractant alfa (N=66)	Statistical Test Value	p-value
Gestational age at birth				
Overall GA (weeks), mean (SD)	28 (2)	26 (2.5)	4.31^	< 0.001
Birth weight				
Overall BW (g), mean (SD)	1070 (366)	875 (378)	3.41^	< 0.001
Gender				
- Male, n/N (%)	71/126 (56.3)	43/66 (65.2)	1.39*	0.238
CRIB II score, mean (SD)	8 (4)	10 (4)	4.7^	< 0.001
Apgar score				
1 min, mean (SD)	5 (2)	5 (2)	0.48^	0.645
5 min, mean (SD)	7 (1)	7 (1.5)	1.54^	0.114
10 min, mean (SD)	8 (1)	8 (1)	1.34^	0.188
Mode of delivery				
- Caesarean section, n/N (%)	34/126 (27)	24/66 (36.4)	1.80*	0.179
- Vaginal delivery, n/N (%)	92/126 (73)	42/66 (63.6)		
Antenatal Steroids				
- No or Inadequate course, n/N (%)	62/126 (49.2)	28/66 (42.4)	0.80*	0.371
- Complete course, n/N (%)	64/126 (50.8)	38/66 (57.6)		
Antenatal Antibiotics				
- No or Inadequate, n/N (%)	90/126 (71.4)	46/66 (69.7%)	0.06*	0.802
- Adequate, n/N (%)	36/126 (28.6)	20/66 (30.3%)		
Antenatal Magnesium Sulphate, n/N (%)	56/126 (44.4)	30/126 (45.5)	0.01*	0.894
Postnatal steroids, n/N (%)	16/126 (12.7)	6/66(9.1)	0.55*	0.456
Positive Maternal Group B streptococcus, n (%)	18/76 (23.7)	11/32 (34.4)	1.31*	0.252
Chorioamnionitis, n/N (%)	8/125 (6.4)	11/66 (16.7)	5.08*	0.024
Premature rupture of membranes, n/N (%)	39/125 (31.2)	22/65 (33.8)	0.13*	0.711
Maternal hypertension n/N (%)	18/126 (14.3)	14/66 (21.2)	1.49*	0.221
Maternal diabetes mellitus, n (%)	36/126 (28.6)	24/66 (36.4)	1.22*	0.269
Early onset sepsis, n/N (%)	5/126 (4.0)	3/66 (4.5)	0.03*	0.849

Primary outcomes

The occurrence of bronchopulmonary dysplasia (BPD) was not statistically significant, showing rates of 68.7% (n=46) in the Poractant group and 47.5% (n=75) in the Beractant group (p=0.71). Likewise, there was no significant difference in mortality rates between the two groups, with 22.5% (n=18) in the poractant group and 11.8% (n=21) in the beractant group (p=0.33). Furthermore, the combined incidence of BPD or mortality was also not statistically significant, recorded at 53.4% (n=95) for the Poractant group compared to 73.8% (n=59) for the Beractant group (p=0.93) (Table 3).

**Table 3:** Outcomes for the complete cohort of preterm infants p-value < 0.05 is considered significant *Adjusted odds ratio, CI, and p-value were calculated using multivariate logistic regression models CI: Confidence Interval; BPD: Bronchopulmonary dysplasia; PVL: Periventricular leukomalacia; IVH: Intraventricular hemorrhage; NEC: Necrotizing enterocolitis; PDA: Patent ductus arteriosus; ROP: Retinopathy of prematurity

Outcomes	Beractant N=178	Poractant N=80	Adjusted odds ratio*	Confidence Intervals	p-value
Primary Outcomes					
BPD, n/N (%)	75/158 (47.5)	46/67 (68.7)	1.18	0.47, 3.01	0.71
Death, n/N (%)	21/178 (11.8)	18/80 (22.5)	0.55	0.15, 1.78	0.33
BPD or Death, n/N (%)	95/178 (53.4)	59/80 (73.8)	1.03	0.42, 2.58	0.93
Secondary Outcomes					
Surfactant Redosing, n/N (%)	81/178 (45.5)	21/80 (26.2)	0.19	0.11, 0.46	<0.00001
Pulmonary Air Leak, n/N (%)	26/178 (14.6)	7/80 (8.8)	0.36	0.12, 0.95	0.05
Pulmonary Haemorrhage, n/N (%)	15/178 (8.4)	6/80 (7.5)	0.30	0.07, 1.02	0.06
PVL, n/N (%)	2/105 (1.9)	6/49 (12.2)	6.38	0.74, 100	0.11
IVH grade ≥ 3, n/N (%)	6/126 (4.8)	9/66 (13.6)	2.01	0.47, 8.5	0.33
NEC stage ≥ 2, n/N (%)	15/178 (8.4)	10/80 (12.5)	0.40	0.11, 1.12	0.12
significant PDA, n/N (%)	22/126 (17.5)	16/66 (24.2)	0.75	0.28,1.91	0.45
ROP requiring treatment, n/N (%)	6/106 (5.7)	10/50 (20)	0.63	0.07, 3.99	0.64

Secondary Outcomes

**Figure 2:**
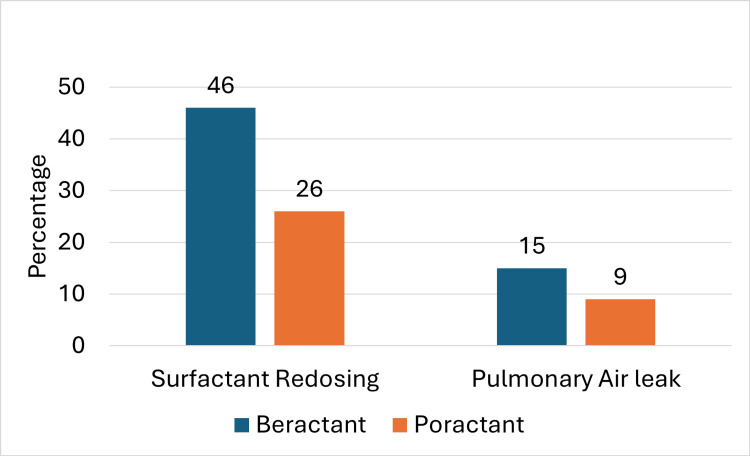
Surfactant redosing and pulmonary air leak distributed in both groups of the whole population.

The need for surfactant redosing was notably lower in the poractant group, with rates of 26.2% (n=21), compared to 45.5% (n=81) in the beractant group (p < 0.00001). Furthermore, pulmonary air leaks occurred less frequently in the poractant group at 8.8% (n=7), while the beractant group had a rate of 14.6% (n=26) (p = 0.05), as shown in Figure 2. No statistically significant differences were found between the two groups for other secondary outcomes. These included pulmonary hemorrhage, periventricular leukomalacia (PVL), intraventricular hemorrhage (IVH), necrotizing enterocolitis (NEC), significant patent ductus arteriosus (PDA), and retinopathy of prematurity (ROP) that required treatment (Table 3).

The outcomes in the subgroup for neonates below 32 weeks of gestation

**Figure 3:**
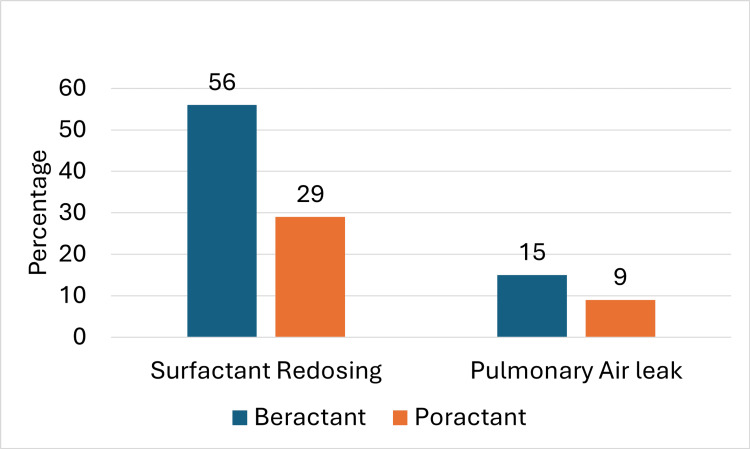
Surfactant redosing and pulmonary air leak distributed in both groups of less than 32 weeks gestational age

Similar to the overall population, the main outcomes, including the incidence of BPD, the mortality rate, and the combined outcome of BPD or mortality, did not show statistical significance after adjusting for confounding factors (Table 4). However, the need for surfactant redosing and the occurrence of pulmonary leak syndrome was significantly lower in the poractant group compared to the beractant group, with rates of 28.8% (n=19) versus 55.6% (n=70) (p < 0.00001) and 9.1% (n=6) versus 15% (n=19) (p = 0.02), respectively, as shown in Figure 3.

**Table 4:** Outcomes for neonates below 32 weeks of gestation p-value < 0.05 is considered significant *Adjusted odds ratio, CI, p-value was calculated using multivariate logistic regression models CI: Confidence Interval; BPD: Bronchopulmonary dysplasia; PVL: Periventricular leukomalacia; IVH: Intraventricular hemorrhage; NEC: Necrotizing enterocolitis; PDA: Patent ductus arteriosus; ROP: Retinopathy of prematurity

Outcomes	Beractant (N=126)	Poractant (N=66)	Adjusted Odds ratio*	Confidence Intervals	P-value
Primary Outcomes					
BPD, n/N (%)	71/106 (67)	45/53 (84.9)	2.16	0.75, 6.87	0.16
Death, n/N (%)	21/126 (16.7)	18/66 (27.3)	0.61	0.20, 1.73	0.37
BPD or Death, n/N (%)	91/126 (72.2)	58/66 (87.9)	1.71	0.62, 5.14	0.30
Secondary Outcomes					
Surfactant Redosing, n/N (%)	70/126 (55.6)	19/66 (28.8)	0.15	0.06, 0.35	< 0.00001
Pulmonary Air Leak, n/N (%)	19/126 (15.1)	6/66 (9.1)	0.25	0.06, 0.80	0.02
Pulmonary Haemorrhage, n/N (%)	15/126 (11.9)	6/66 (9.1)	0.35	0.09, 1.16	0.10
PVL, n/N (%)	2/105 (1.9)	6/49 (12.2)	6.38	0.74, 100	0.11
IVH grade ≥ 3, n/N (%)	6/126 (4.8)	9/66 (13.6)	2.01	0.47, 8.5	0.33
NEC stage ≥ 2, n/N (%)	15/126 (11.9)	10/66 (15.2)	0.45	0.13, 1.40	0.18
significant PDA, n/N (%)	22/126 (17.5)	16/66 (24.2)	0.75	0.28,1.91	0.45
ROP requiring treatment, n/N (%)	6/106 (5.7)	10/50 (20)	0.63	0.07, 3.99	0.64

## Discussion

The primary finding of this study, after adjusting for confounding factors, indicated no statistically significant difference in the incidence of BPD or mortality between the Poractant group and the Beractant group. While the BPD rate was 68.7% (n=46) of infants in the Poractant group versus 47.5% (n=75) in the Beractant group with p=0.71, the mortality rates were present in 22.5% (n=18) and 11.8% (n=21), respectively, p=0.33. These results align with the findings of Lemyre et al. (2022), who also observed no significant difference in survival to discharge and/or BPD between the two groups [11]. Conversely, a recent meta-analysis by Tridente et al. (2019) suggested a potential decrease in mortality along with a statistically significant reduction in the combined outcome of BPD or mortality in neonates receiving a 200 mg/kg dose of Poractant alfa compared to those treated with bovine surfactant [3].

Additionally, maternal and infant demographic factors played a crucial role in our study outcomes. The higher incidence of chorioamnionitis in the Poractant group, 15% (n=12) vs. 4.5% (n=8) in the Beractant group, may have contributed to the observed differences in BPD and mortality rates. This was highlighted by Sánchez Luna et al. (2020), who noted that antenatal factors significantly impact neonatal outcomes. Furthermore, the Poractant group had a higher proportion of extremely preterm and low birth weight infants, which may have influenced the primary outcomes. Numerous studies indicate that gestational age and birth weight are important predictors of neonatal outcomes, regardless of the surfactant used [17-19].

Our study demonstrated that the Poractant group had a significantly lower need for surfactant redosing 26.2% (n=21) vs. 45.5% (n=81) in the Beractant arm, p<0.05. A recent study also confirmed that Poractant alfa significantly reduced the requirement for additional doses of surfactant in preterm infants with RDS [20]. This finding aligns with earlier research which indicated that a lower percentage of patients in the Poractant alfa group required two or more doses compared to the beractant group 31% versus 12%, p = 0.023 [21]. The decreased need for surfactant redosing in the Poractant group raises potential benefits in lowering the frequency of interventions and related problems.

Furthermore, this study showed a lower incidence of air leak syndrome in the poractant group, 14.6% (n=26) vs. 8.8% (n=7) in the beractant arm, p=0.05. A recent retrospective cohort study published in 2024 found that the difference in the incidence of air leak syndrome between infants treated with Poractant alfa and those treated with beractant was statistically insignificant (p = 0.536) [22]. On the contrary, a meta highlighted a lower incidence of air leak syndrome between Poractant alfa and Beractant (p = 0.006) [3].

In our study, the occurrence of other outcomes, such as significant PDA, IVH, ROP, PVL, NEC, was similar in both groups. Multiple studies have consistently shown no significant differences in the incidence rates of PDA, IVH, PVL, ROP, and NEC between infants treated with Poractant and those treated with Beractant [23-26].

Strengths and limitations

To our knowledge, our study is among the first in our region to evaluate the short-term outcomes of Poractant versus Beractant surfactant. Nevertheless, our study's limitations include retrospective data collection from medical records, a small sample size reflecting a single-center experience, variation in patient demographic characteristics, and evaluation of the short-term outcomes only with little attention to long-term or neurodevelopment outcomes. Therefore, our data should be interpreted with caution.

## Conclusions

There was no significant difference in the rates of bronchopulmonary dysplasia or mortality between Poractant alfa (CUROSURF®) and Beractant (SURVANTA®) in preterm infants suffering from respiratory distress syndrome. Poractant alfa (CUROSURF®) showed a reduced need for surfactant redosing and a lower incidence of air leak syndrome. However, the rates of other outcomes, including significant patent ductus arteriosus (PDA), intraventricular hemorrhage (IVH), retinopathy of prematurity (ROP), periventricular leukomalacia (PVL), and necrotizing enterocolitis (NEC), were comparable in both treatment groups. Further randomized prospective studies are necessary to evaluate these types of surfactants and investigate their efficacy and both short- and long-term outcomes.
